# Evidence for Association of Cell Adhesion Molecules Pathway and *NLGN1* Polymorphisms with Schizophrenia in Chinese Han Population

**DOI:** 10.1371/journal.pone.0144719

**Published:** 2015-12-16

**Authors:** Zhengrong Zhang, Hao Yu, Sisi Jiang, Jinmin Liao, Tianlan Lu, Lifang Wang, Dai Zhang, Weihua Yue

**Affiliations:** 1 Institute of Mental Health, The Sixth Hospital, Peking University, Beijing, China; 2 Key Laboratory of Mental Health, Ministry of Health & National Clinical Research Center for Mental Disorders (Peking University), Beijing, China; 3 School of Life Sciences, Tsinghua University, Beijing, China; 4 Tsinghua University-Peking University Joint Center for Life Sciences, Beijing, China; 5 PKU-IDG/McGovern Institute for Brain Research, Peking University, Beijing, China; Chiba University Center for Forensic Mental Health, JAPAN

## Abstract

Multiple risk variants of schizophrenia have been identified by Genome-wide association studies (GWAS). As a complement for GWAS, previous pathway-based analysis has indicated that cell adhesion molecules (CAMs) pathway might be involved in the pathogenesis of schizophrenia. However, less replication studies have been reported. Our objective was to investigate the association between CAMs pathway and schizophrenia in the Chinese Han population. We first performed a pathway analysis utilizing our previous GWAS data. The CAMs pathway (hsa04514) was significantly associated with schizophrenia using hybrid gene set-based test (*P* = 1.03×10^−10^) and hypergeometric test (*P* = 5.04×10^−6^). Moreover, 12 genes (*HLA-A*, *HLA-C*, *HLA-DOB*, *HLA-DPB1*, *HLA-DQA2*, *HLA-DRB1*, *MPZ*, *CD276*, *NLGN1*, *NRCAM*, *CLDN1* and *ICAM3*) were modestly significantly associated with schizophrenia (*P*<0.01). Then, we selected one promising gene neuroligin 1 (*NLGN1*) to further investigate the association between eight significant SNPs and schizophrenia in an independent sample (1814 schizophrenia cases and 1487 healthy controls). Our study showed that seven SNPs of *NLGN1* and two haplotype blocks were significantly associated with schizophrenia. This association was confirmed by the results of combined analysis. Among them, SNP rs9835385 had the most significant association with schizophrenia (*P* = 2.83×10^−7^). Furthermore, in silico analysis we demonstrated that *NLGN1* is preferentially expressed in human brain and SNP rs1488547 was related to the expression level. We validated the association of CAMs pathway with schizophrenia in pathway-level and identified one susceptibility gene *NLGN1*. Further investigation of the roles of CAMs pathway in the pathogenesis of schizophrenia is warranted.

## Introduction

Schizophrenia is a server and complex psychiatric disorder with an estimated worldwide-pooled prevalence of 1%, characterized by hallucinations, delusions, disturbed emotions and social withdrawal [1, 2]. Decades of research for the biological pathogenesis of schizophrenia still did not completely elucidate the clearly causes of this disorder. Given its high heritability of over 80% [3], exploration of genetic mechanisms involved in schizophrenia has been attracted more and more attention [4]. Recent researches have considerably advanced our understanding in terms of identifying risk loci and the mechanisms by which genetic risk is conferred [5, 6]. Genome-wide association studies (GWAS) have identified some promising candidate genes association with schizophrenia, such as *DRD2*, *TCF4*, *NOTCH4*, *CACNA1C* and *ZNF804A* [7–10]. However the evidence of involvement of specific genes and variants remained elusive.

The traditional GWAS typically investigated the genetic effect of one Single Nucleotide Polymorphism (SNP) at a time and it accounted for only a small proportion of the heritability of schizophrenia, leaving a large portion of the disease’s susceptibility unexplained [11]. There is growing evidence that undetected association may partially reside in genetic variation with lower odds ratios. Furthermore, most common diseases are multigenic traits which involve a group of genes functioning at various stages of disease development [12]. Therefore, pathway-based methods were developed to complement the original analysis of GWAS. The basic idea of these methods is that disease association of a pathway may become prominent via an excess of (moderately) significant SNPs belonging to the genes of the pathway. Combined with GWAS data, these pathway-based approaches could assess whether a group of genes or pathways with related functions are jointly associated with a trait of interest and generate specific hypothesis for follow-up experimental studies [12]. The small effects of some variants may be discovered through this method, so the pathway analysis might explain the missing heritability in GWAS and provide novel insights into etiology of complex diseases. Recently, utilizing schizophrenia GWAS data and pathway datasets, the researchers have reported several pathway to be significantly associated with schizophrenia, such as cell adhesion molecules pathway, ion channels pathways [13], myelin-related pathways [14], glutamate metabolism pathway, TGF-beta signaling pathway and TNFR1 pathway [15]. It was noteworthy that the cell adhesion molecules (CAMs) pathway proved to be significantly associated with both schizophrenia and bipolar disorder in European population [16]. The CAMs pathway was also found to have a critical role in cognition function including memory formation and attention-related reaction time [17]. Additionally, previous researches have shown that altered neuronal precise adhesion connections during the development of the human nervous system could contribute to dysfunction of neural circuits, which may constitute the basis the etiology of numerous cases of neurological disorders [18].

Cell adhesion molecules are glyco proteins expressed on the cell surface and play a critical role in a wide array of biologic processes that include the immune response, inflammation, embryogenesis, and development of neuronal tissue. There were four main groups: the integrin family, the immunoglobulin superfamily, selectins and cadherins [19]. Cell-cell adhesions are crucial for neuron morphology, cell signal, axon guidance, synaptic plasticity, myelination and highly coordinated brain functions including memory and learning [20]. Cell adhesion molecule genes which connect neurons with others are crucial for involvement in synapse formation, synaptic plasticity and cell signals [21]. These promising candidate genes have been reported about biological and genetic mechanism. For example, the proteins with abnormal NCAM/synaptic ratios have been reported in the hippocampus and cingulate cortex of schizophrenia patients [22, 23]. Neurexins and neuroligins from one of the most studies molecular contributing to synaptic connection have been reported in patients with schizophrenia including neurexin-1, and neuroligin-2 [24, 25]. In addition, the GWAS has identified immune-related major histocompatibility complex (MHC) SNPs association with schizophrenia [26]. Mutations or aberrant expression of CAMs may lead to changes in synaptic morphology and function and are associated with many neurological disorders [17]. The existing researches supported the role of CAMs pathway in neurodevelopment psychiatry, both at the level of individual SNPs and the wider pathway. However, the roles of the CAMs pathway in schizophrenia in Chinese Han population are rarely known [16, 17]. Among the genes of the CAMs pathway, the neuroligin 1 (*NLGN1*)gene aroused our interest. Recently, we carried out a GWAS of schizophrenia in the Chinese Han population [27] and found several novel candidate genes associated with schizophrenia, including the *NLGN1* gene. In a recent Psychiatric GWAS Consortium (PGC) meta-analysis of schizophrenia GWAS (36,989 cases and 113,075 controls) [7], there were several SNPs in the *NLGN1* polymorphism that also showed moderate association with schizophrenia. Furthermore, NLGN1 is a postsynaptic protein binding to presynaptic neurexin and dynamically shapes excitatory synaptic efficacy and plasticity, participating in the formation of long-term memory [28–30]. The *NLGN1* gene was previously reported to have significant association with other psychiatric disorders such as autism and major recurrent depression, which share some clinical symptoms with schizophrenia [31, 32].

Here we examined whether the CAMs pathway and *NLGN1* contributed to schizophrenia susceptibility in Chinese Han population. First, we performed pathway-based analysis by applying the hybrid gene set-based test (HYST) and hypergeometric test to our previous GWAS data. Among several associated genes discovered in pathway analysis, we validated the gene *NLGN1* which is a significant adhesion molecular. Then, to corroborate the association between the *NLGN1* gene and schizophrenia in the Chinese Han population, we performed an independent replication study in 1814 schizophrenia cases and 1487 healthy controls. We also performed a meta-analysis using this replication sample and the first-stage GWAS sample and further investigate the relationship between *NLGN1* and schizophrenia in silico analysis.

## Methods

### 2.1 GWAS data of Schizophrenia in Chinese Han population

The schizophrenia GWAS data was from our previous study [27]. Our GWAS samples (768 schizophrenia cases and 1,733 normal controls) came from individuals of Han Chinese ancestry, genotyped with Illumina Human610-Quad BeadChips. Individual quality control was based on the following criteria: we excluded samples with poor genotyping or relative relationship, or the samples which were population outliers. SNP quality control was based on the following criteria: we removed SNPs with call rates less than 90%, SNPs with minor allele frequency (MAF) less than 5%, and SNPs with significant deviation from Hardy-Weinberg equilibrium (HWE) in controls (*P*<1×10^−5^). Finally, a total of 448,734 autosomal SNPs in 746 schizophrenia cases and 1599 normal controls were retained. Detailed sample information, genotyping quality control, genomic control, and statistical analyses were provided in the in our previous study [27].

### 2.2 Pathway Analysis

First, we extracted pathway data from the canonical pathway database, Kyoto Encyclopedia of Genes and Genomes (KEGG) (http://www.kegg.jp/kegg/pathway.html) and obtained 133 genes involved in cell adhesion molecule (hsa04514). Then, we used pathway-based analysis software ‘KGG’ developed by Li et al [33]. The KGG software consisted of three main steps. First, the genotyped SNPs of our previous GWAS in 133 CAMs genes were mapped to their respective genes based on their coordinates from the RefSeq gene annotations database (hg18). We also included the region 5 kb upstream and 5 kb downstream of each gene to account for variants in potential promoter regions. Second, KGG combined SNP-based P-values with estimated of linkage disequilibrium among SNPs within the gene to provide a gene-based P-value, using Gene-Based Association Test (GATES) [34]. The computer simulations demonstrated that this test offers effective control of the type 1 error rate regardless of gene size and linkage disequilibrium pattern among markers [34]. The statistical power in simulated data is at least comparable, and often superior, to that of several alternative gene-based tests [34]. Third, the pathway-level statistics were calculated by combining all gene-based p-values for association, using the HYST method, a combination of a scaled *χ* ^2^-test [33] and an extended Simes test [34] that integrates gene-based P-values to obtain an overall P-value for the entire pathway. To explain the logic of the study design, a flow chart summarizing the analytical methods was shown in S1 Fig.

### 2.3 Gene selection

The sample size of our previous GWAS was relatively small (746 schizophrenia cases and 1,599 controls), so we intend to validate one promising gene in SNP level. We intended to select one promising gene among these genes which were nominally associated schizophrenia (gene p-value<0.01) and contributed much to the association of the CAMs pathway. To further explore the association between the selected gene and schizophrenia, we extracted the SNP data of this gene from the public PGC schizophrenia GWAS (published in 2014), which included 35,476 schizophrenia cases and 46,839 controls (http://www.med.unc.edu/pgc) [7]. Then, we performed the gene-based test with these SNPs data using the KGG software.

### 2.4 The replication study

To further confirm the association results of the selected gene in Chinese Han population, we selected SNPs with P-value<0.01 in this gene and performed an independent replication study in 1814 cases (878 males and 936 females; mean age: 30.1±10.7 ears) and 1487 controls (707 males and 780 females; mean age: 29.7±9.8 years). This sample was fully independent of the samples described in our previous GWAS and its validation study. All subjects were of Chinese Han ethnicity, and were born and residing in China. The consensus diagnoses were made by at least two experienced senior psychiatrists according to the Diagnosis and Statistic Manual of Mental Disorders, 4th edition (DSM-IV) criteria for schizophrenia. Patients with previously diagnosed diabetes, thyroid disease, hypertension, heart disease and other severe physical diseases were excluded. Healthy controls were recruited from communities with simple non-structured interviews performed by psychiatrists, who excluded individuals with any history of mental health and/or neurological disorder. Controls were matched with patients on location of their residence, gender and age. They gave written informed consent for the genetic study, which was approved by the Ethical Committee of Institute of Mental Health, Peking University.

Peripheral blood samples were collected from all subjects. Genomic DNA was extracted from the blood using the Qiagen QIAamp DNA Mini Kit (Qiagen). Single SNPs were genotyped using TaqMan SNP genotyping assay on an ABI PRISM 7900 Sequence Detection Systems (Applied Biosystems, FosterCity, CA). PCR was performed following the standard protocol with 5 μl reaction volumes for each well in a 384-well plate and contained 5 ng of DNA. The thermal cycling conditions were 1 cycle at 95°C for 10 min, 50 cycles of 92°C for 15 s, and 60°C for 1 min. The Sequence Detection System (SDS) Version 2.0 software (Applied Biosystems) was used for genotypic identification. For quality control purposes, all genotypes were blind to the case or control during the genotyping process. We repeated the genotyping assay for 1% of the samples and found no inconsistency. The DNA extraction and genotyping were centrally processed at the Key Laboratory of Mental Health in Beijing.

The Haploview program (version 4.1) was applied to test the genotypic distributions of SNPs for HWE, to estimate linkage disequilibrium (LD) between paired SNPs using the *r* ^*2*^ algorithm, and to determine the haplotype blocks. Haplotype blocks were defined according to the criteria of Gabriel et al [35].

For the meta-analysis, heterogeneity across the two samples was evaluated using the Cochran Q statistic to determine the heterogeneity statistic (*I* ^*2*^) and P value. The meta-analysis were made by using PLINK [36]. Generally, *I* ^*2*^<30% signifies no heterogeneity, *I* ^*2*^ = 30~50% signifies moderate heterogeneity, and *I* ^*2*^>50% indicates strong heterogeneity. If *I* ^*2*^<50%, the fixed-effect model was used to combine the results from the two different cohorts; otherwise, the random-effect model was used [37, 38].

### 2.5 Gene expression analysis in human tissues

To explore the expression pattern of the selected gene in human tissues, we used two different sets of expression data. First, we investigated the tissue-specific expression distributions of this gene in human tissues in Gene Enrichment Profiler (http://xavierlab2.mgh.harvard.edu/EnrichmentProfiler/). In this database, the expression enrichment of any set of query genes was computed on the basis of a reference set obtained from 126 normal tissues and cell types [39]. The gene expression intensities have been converted to enrichment scores, which reflect the enrichment of a gene based on its expression in all tissues. For each tissue, the enrichment scores of the gene were compared to the other genes in the genome with a one-tailed rank-sum test, and the p-value for each tissue was plotted. Then, we investigated the expression pattern of *NLGN1* in brain regions by utilizing the Human Brain Transcriptome (HBT) (http://hbatlas.org/pages/hbtd) [40]. The HBT database includes transcriptome of 16 regions comprising the cerebellar cortex, mediodorsal nucleus of the thalamus, striatum, amygdala, hippocampus, and 11 areas of the neocortex. Totally, 1,340 tissue samples were collected from 57 developing and adult post-mortem brains.

### 2.6 Expression quantitative trait loci (eQTL) analysis

It was identified that an enrichment of eQTLs amongst human complex disease GWAS loci, suggesting that eQTLs may play an important role in disease-associated variants identified in GWAS. [41, 42] Furthermore, previous studied suggested that changes in gene expression played a key role in the pathogenesis of schizophrenia. [43, 44] To detect the functional effects of the risk SNPs in the selected gene, we analyzed their associations with gene expression levels in two independent data sets: Genevar and Braineac. Detailed processing and exclusion criteria for both data sets are described elsewhere [45, 46]. In brief, among several datasets of Genevar, we used one data set from Dimas et al. [47], which correlated genome-wide gene expression in lymphoblastoid cell lines from a total of 75 individuals. Moreover, the Braineac Database (http://www.braineac.org/) consisted of 134 neuropathologically normal donors from the MRC Sudden Death Brain Bank in Edinburgh and Sun Health Research Institute; expression was profiled on the Affymetrix Exon 1.0 ST array. In Braineac database, we can examine generated eQTL data for ten human brain regions (cerebellar cortex, frontal cortex, hippocampus, inferior olivary nucleus, occipital cortex, putamen, substantia nigra, temporal cortex, thalamus and intralobular white matter). Furthermore, to find weaker but ubiquitous signals in the human brain, the mean expression profile was also calculated across the ten brain regions [46].

## Results

### 3.1 Pathway analysis of the GWAS data

**Fig 1:**
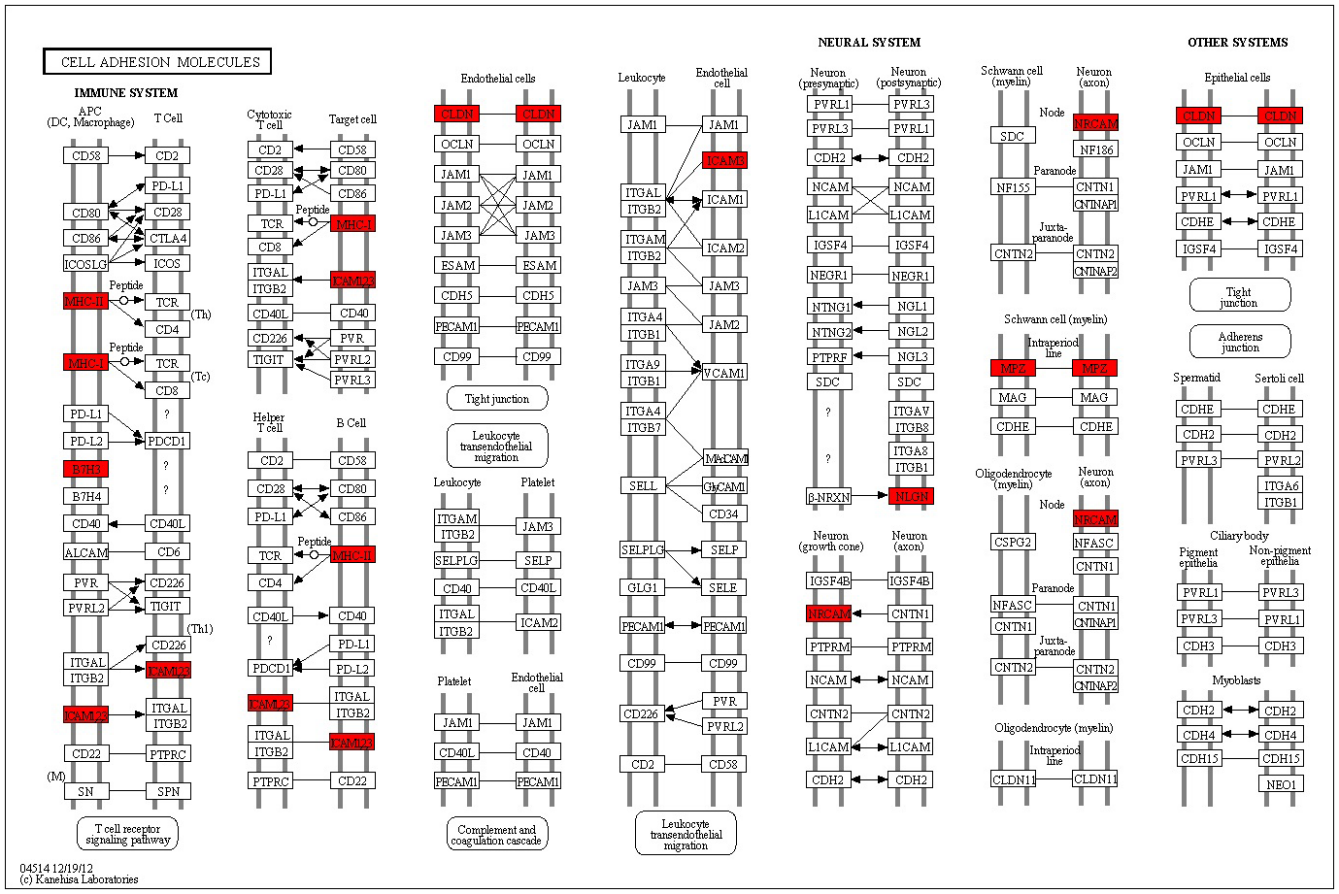
Genes involved in ‘cell adhesion molecules’ pathway. Color scheme: Red = significant genes (*P*<0.01). White = non-significant genes. See detail in (http://www.genome.jp/kegg-bin/show_pathway?hsa04514).

To test whether cell adhesion molecules (CAMs) pathway was critical to schizophrenia, we conducted a pathway analysis using our previous GWAS data with KGG software. In total, 3,712 SNPs in our previous GWAS were mapped to 133 genes in the cell adhesion molecular (CAMs) pathway. In pathway level, the CAMs pathway was significant associated with schizophrenia using hybrid gene set-based test (*P* = 1.03×10^−10^) and hypergeometric test (*P* = 5.04×10^−6^). In addition, it was also important for pathway analysis to examine the SNPs and genes results. In SNP level, 302 SNPs had P-values less than 0.05. There was no SNP achieved the genome-wide significance level (*P*<5×10^−8^). In gene level, 12 genes (*HLA-A*, *HLA-C*, *HLA-DOB*, *HLA-DPB1*, *HLA-DQA2*, *HLA-DRB1*, *MPZ*, *CD276*, *NLGN1*, *NRCAM*, *CLDN1* and *ICAM3*) were modestly significantly associated with schizophrenia (*P*<0.01). However, no genes survived after the Bonferroni correction (significantly corrected *P*< 0.00038, i.e. α = 0.05/133). The results of gene level were shown in Table 1 and all the twelve genes were mapped to CAMs pathway in Fig 1.

**Table 1:** Significant associated genes involved in cell adhesion moleculars pathway.

Gene	Chr	Start Position	Size (kb)	*P*-value	S ^a^	No. of SNPs ^b^	Function
*MPZ*	1	161269525	15.24	0.0038	4	6	Peripheral myelin protein
*NLGN1*	3	190018490	894.87	0.0056	27	193	Formation and remodeling of central nervous system synapses
*CLDN1*	3	173111244	26.75	0.0065	5	9	Integral membrane protein and tight junction strands component
*HLA-A*	6	29905309	13.35	0.0006	2	8	HLA class I heavy chain paralogues
*HLA-DPB1*	6	31231529	21.22	0.0031	10	15	HLA class II beta chain paralogues
*HLA-DOB*	6	32704163	14.29	0.0046	6	21	HLA class II beta chain paralogues
*HLA-C*	6	32541546	13.33	0.0061	8	20	HLA class I heavy chain paralogues
*HLA-DQA2*	6	33038760	15.5	0.0071	6	20	HLA class II alpha chain family
*HLA-DRB1*	6	32775540	21.02	0.0083	2	3	HLA class II beta chain paralogs
*NRCAM*	7	107783071	318.77	0.0046	19	81	Neuron-neuron adhesion and signaling transduction
*CD276*	15	73971622	40.24	0.0048	3	6	Regulation of T-cell-mediated immune response
*ICAM3*	19	10439452	15.89	0.0093	1	10	Adhesion and signaling molecule

Chr chromosome; kb, kilobase; SNP, single-nucleotide polymorphism;

^a^ The number of significant SNPs *(P*<0.05) in gene;

^b^ The number of SNPs in corresponding gene from our GWAS data.

### 3.2 Gene selection

Among the 12 genes which contributed much to the association of the CAMs pathway and were nominally associated schizophrenia (gene p-value<0.01), we focused on the *NLGN1* gene, because it was previously identified to be associated with other psychiatric disorders, including autism and bipolar disorder [31, 32] and it was involved in many important brain functions such as synaptic plasticity and long-term memory [28–30]. However, to our knowledge, the role of NLGN1 for schizophrenia was rarely known. To explore the association between *NLGN1* and schizophrenia, we extracted the SNP data of *NLGN1* from our previous GWAS and the public PGC schizophrenia GWAS, which included 35,476 schizophrenia cases and 46,839 controls. Then, we performed the gene-based test using the GATES method. In SNP level, 27 SNPs of the *NLGN1* gene were nominally associated with schizophrenia (*P*<0.05) in our previous GWAS (the most significant SNP rs6792822, *P* = 7.18×10^−5^) and 3 SNPs in PGC schizophrenia GWAS achieved the suggestive significance level (*P*<1×10^−6^, the most significant SNP rs34626435, *P* = 3.32×10^−7^) (S1 Table). In gene-level, the *NLGN1* gene was also significant associated with schizophrenia in our dataset (*P* = 5.6×10^−3^) and PGC dataset (*P* = 6.72×10^−4^). The results of SNP and gene level from these two GWAS suggested that *NLGN1* might be associated with schizophrenia.

### 3.3 Common Variants (MAF>0.05) in *NLGN1* Gene were Significantly Associated with Schizophrenia

To further validate the association of *NLGN1* in Chinese Han population, we selected eight most significant SNPs (*P*<0.01) of *NLGN1* gene in our previous GWAS to validate in 1,814 schizophrenia cases and 1,457 healthy controls. All of the eight SNPs we selected showed MAFs greater than 5% in our samples. The genotype distributions of the eight SNPs in the case/control group did not show significant deviations from Hardy-Weinberg equilibrium. No significant differences in age or gender distributions were found between the case and control samples. Six SNPs remained significant with schizophrenia after the Bonferroni correction (significantly corrected *P*< 0.006, i.e. α = 0.05/8) which was considered as a conservative correction method (Table 2).

**Table 2:** Results of single marker association for the SNPs in *NLGN1* gene.

Markers	Pos.	Allele	Previous GWAS data^a^				Replication study^a^					Combined analysis	
			MAF		*P*-value	OR	MAF		*P-*value	OR			
			Case	Control			Case	Control			*I* ^*2*^	*P* values	OR
rs13074723	173522097	A/G	0.221	0.186	0.0049	1.242	0.214	0.185	0.0033	1.200	0	4.98E-05	1.2164
rs1488547	173525768	G/A	0.221	0.188	0.0085	1.225	0.216	0.186	0.0030	1.202	0	7.32E-05	1.211
rs2861598	173544812	G/A	0.221	0.184	0.0035	1.253	0.213	0.183	0.0021	1.211	0	2.35E-05	1.2274
rs4280663	173623133	G/A	0.218	0.178	0.0011	1.289	0.220	0.190	0.0028	1.202	0	1.24E-05	1.2349
rs4399918	173604333	G/A	0.418	0.464	0.0035	0.831	0.427	0.450	0.0609	0.911	21.99	1.05E-03	0.8795
rs4513478	173626250	G/A	0.429	0.387	0.0057	1.192	0.422	0.386	0.0026	1.164	0	4.53E-05	1.1747
rs9835385	173602528	A/G	0.215	0.172	4.43E-04	1.317	0.216	0.179	0.0002	1.265	0	2.83E-07	1.2849
rs6792822	190030324	T/C	0.298	0.243	7.18E-5	1.32	0.284	0.270	0.2009	1.073	73.87	4.39E-03	1.1295

Pos., position; Allele, minor allele/major allele; MAF, minor allele frequency; OR, odds ratio; *I* ^*2*^, heterogeneity test.

^a^ The sample of previous GWAS enrolled 746 cases and 1,599 controls. The sample of current replication study included 1,814 case and 1,457 controls.

**Fig 2:**
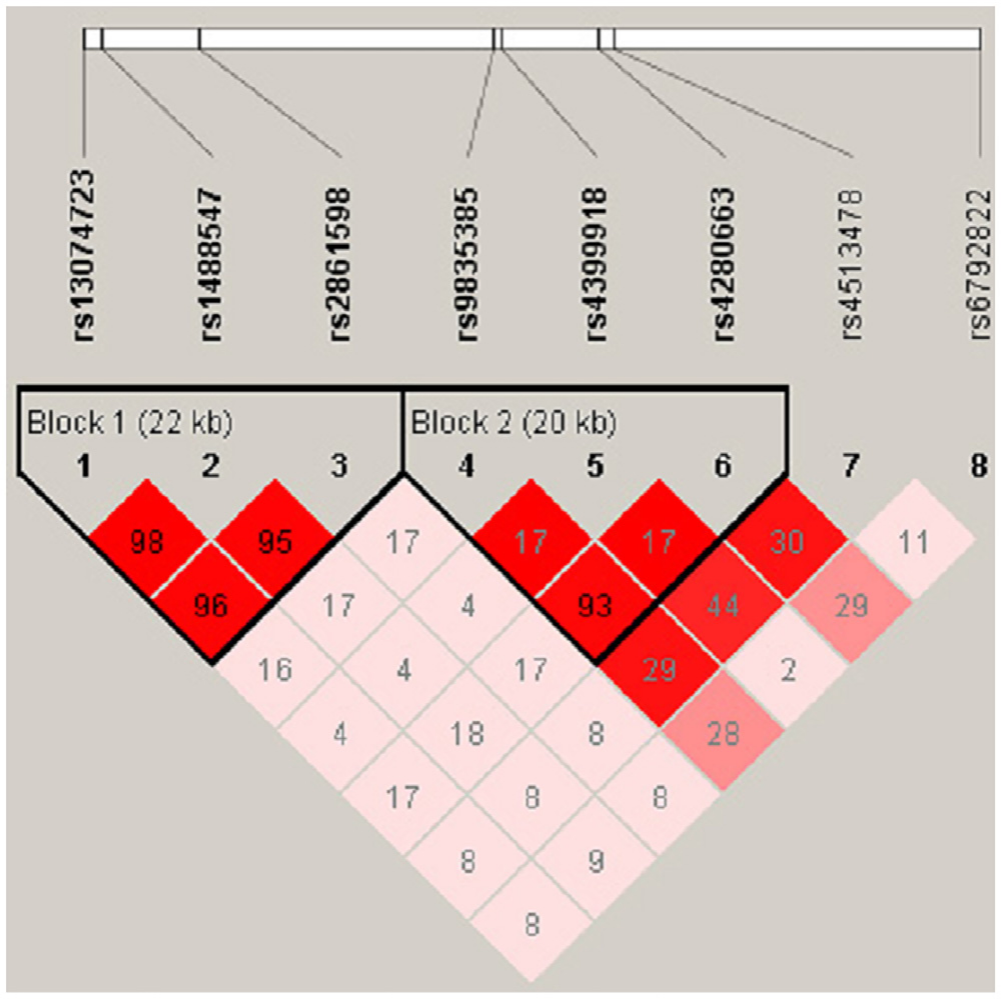
Linkage disequilibrium of the seven significant SNPs identified in this study. The LD pattern was derived from the combined group (i.e., both case and healthy control subjects in our previous GWAS). The LD block was defined according to the criteria of Gabriel et al (38). The color scale ranges from red to white (color intensity decreases with decreasing *r* ^*2*^ value). This plot was generated by Haploview (version 4.1).

To analyze the haplotype structure of the eight SNPs, linkage disequilibrium was computed between every two SNPs. We found that six of eight significant SNPs are located in 2 haplotype blocks (Fig 2). Then, to determine whether any specific haplotypes would confer a higher risk for schizophrenia, the specific and global haplotype association tests were performed. Three risk haplotypes were significantly associated with schizophrenia. The haplotypes G-A-A (rs13074723-rs1488547-rs2861598), A-G-G (rs13074723-rs1488547-rs2861598) and A-A-G (rs9835385-rs4399918-rs4280663) showed nominal differences between the patient and control groups. The results were still significant after the 10,000-time permutation tests. S2 Table showed the results of haplotype-based association test using the same eight SNPs based on the previous GWAS data. The results of the present study and previous GWAS were highly consistent (S3 Table).

Then, we combined the results of this replication study with our GWAS data (Table 2). In our combined analysis, we used the fixed-effect model to combine the results from the two different cohorts. In the combined analysis, except for SNP rs6792822, the other seven SNPs still had strong associations with schizophrenia and passed heterogeneity test (*I* ^*2*^<30). After Bonferroni correction, seven SNPs with no heterogeneity remained significant between cases and controls (Table 2). This result supported that *NLGN1* might confer risk for schizophrenia.

### 3.4 Expression profiling of the *NLGN1* gene in the Human Tissues

**Fig 3:**
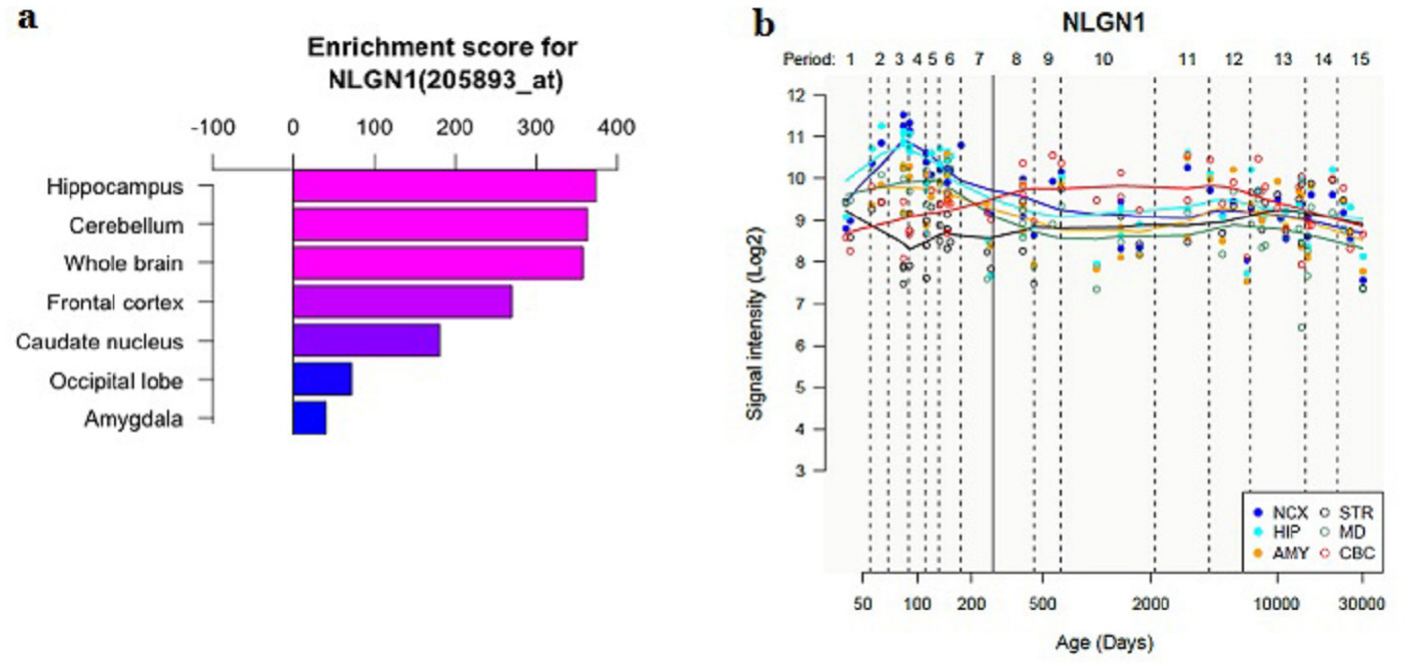
Temporal-spatial expression profiling of *NLGN1* in human brain tissues. (a) Expression of *NLGN1* is enriched in human brain tissues, the hippocampus, cerebellum, whole brain and frontal cortex have the highest enrichment scores. (b) Temporal expression pattern of *NLGN1* in different human brain regions. AMY, amygdala; CBC, cerebellar cortex; HIP, hippocampus; MD, mediodorsal nucleus of the thalamus; NCX, neocortex; STR, striatum. Periods of human development and adulthood as defined in this figure: Embryonic (periods 1), Early fetal Early mid-fetal (periods 2–3), Early mid-fetal (periods 3–4), Late mid-fetal (periods 6), Late fetal (periods 7), Neonatal and early infancy (periods 8), Late infancy (periods 9), Early childhood (periods 10), Middle and late childhood (periods 11), Adolescence (periods 12), Young adulthood (periods 13), Middle adulthood (periods 14), Late adulthood (periods 15).

To test the biological plausibility of *NLGN1* in the pathogenesis of schizophrenia, we investigated expression enrichment profiling of the *NLGN1* gene in multiple human tissues. We found the *NLGN1* was preferentially expressed in human brain tissues: the hippocampus, cerebellum, whole brain and frontal cortex have the highest enrichment scores (Fig 3). Temporal expression analysis showed that the expression level of *NLGN1* was relatively high at early developmental stages (fetal age), as development continued, the expression of the *NLGN1* was gradually decreased in human brain (Fig 3). The results of gene expression analysis further supported the potential role of *NLGN1* in brain function and schizophrenia susceptibility.

### 3.5 SNP rs1488547 was associated with the expression level of *NLGN1* mRNA

**Fig 4:**
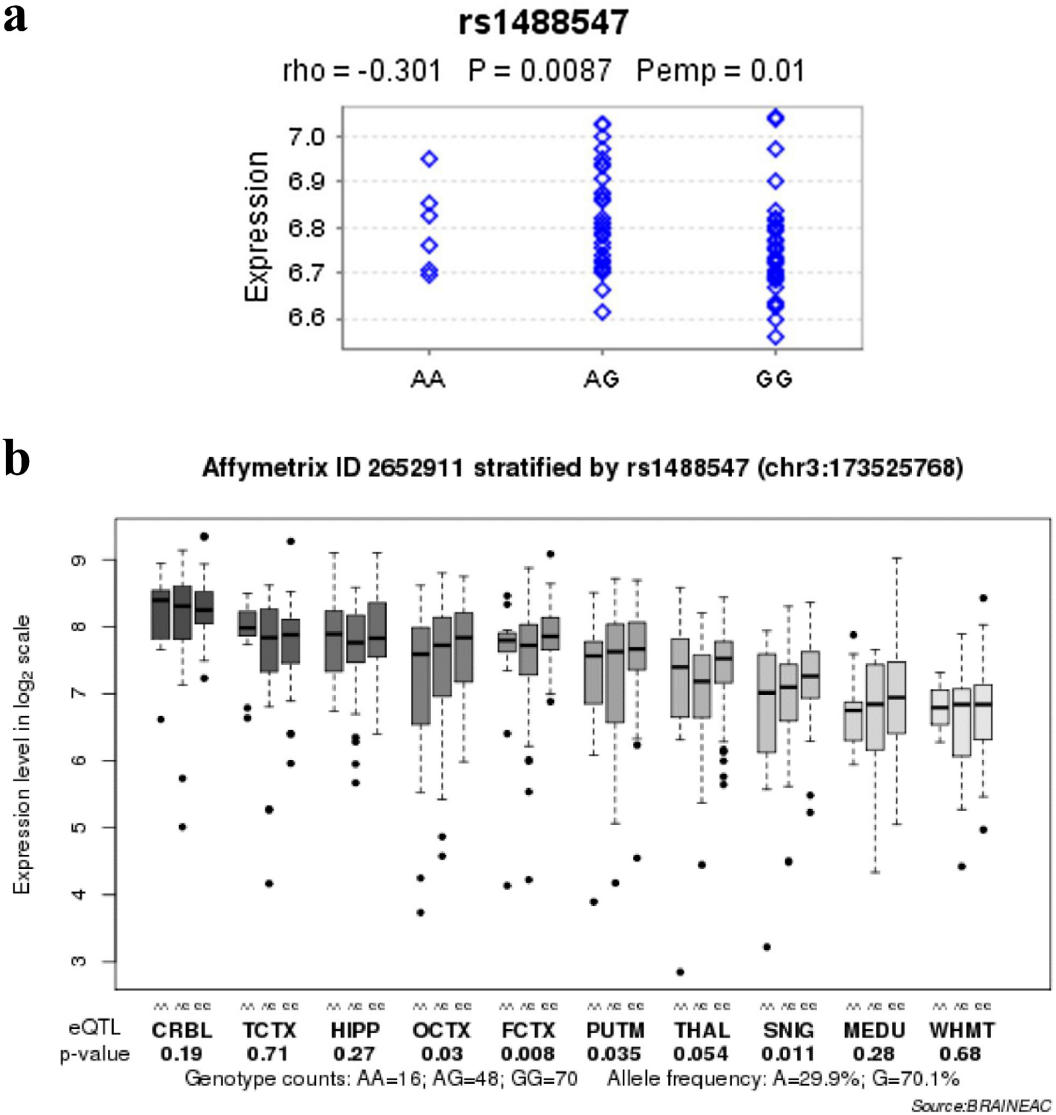
Effects of risk single-nucleotide polymorphisms on *NLGN1* mRNA expression. (a) Results in the lymphoblastoid cell lines of 75 healthy subjects. (b) Results in ten brain regions of 134 healthy subjects. SNIG, substantia nigra; MEDU, the inferior olivary nucleus (sub-dissected from the medulla); THAL, thalamus; WHMT, intralobular white matter; TCTX, temporal cortex; HIPP, hippocampus; PUTM, putamen; FCTX, frontal cortex; OCTX, occipital cortex; CRBL, cerebellar cortex.

To examine the association between six significant SNPs and *NLGN1* expression, we first used the Genevar database. We found rs1488547, which located in intron 3 of *NLGN1*, was nominally associated with the expression level of *NLGN1* in lymphoblastoid cell lines (*P* = 0.0087) (Fig 4). However, it did not survive after Bonferroni correction (significantly corrected *P*< 0.0083, i.e. α = 0.05/6, where 6 was the number of tests carried out: 6 SNPs for *NLGN1*). To further explore the association between the six identified SNPs and *NLGN1* expression in brain, we first investigated the associations between and the expression level of *NLGN1* in Braineac database. We found rs1488547 was also significantly associated with the mean expression level of *NLGN1* across the available brain regions of normal subjects (*P* = 0.0072) and survived after Bonferroni correction (significantly corrected *P*< 0.0083, i.e. α = 0.05/6, where 6 was the number of tests carried out: 6 SNPs for *NLGN1*). We hypothesized that the SNP rs1488547 might be associated with the expression level of *NLGN1* in a specific brain region. Therefore, we further investigated the association between rs1488547 and *NLGN1* expression in ten brain regions respectively. The SNP rs1488547 also showed nominal association with the expression of *NLGN1* in frontal cortex (*P* = 0.008), putamen (*P* = 0.035), occipital cortex (*P* = 0.03) and substantia nigra (*P* = 0.011) (Fig 4). However, it did not survive after Bonferroni correction (significantly corrected *P*< 0.005, i.e. α = 0.05/10, where 10 was the number of tests carried out: 10 brain regions for rs1488547).

## Discussion

Schizophrenia is a complex psychotic disorder that affects millions of individuals worldwide. Recent genetic studies have vastly promoted the progress of schizophrenia research. Although many promising candidate genes have been identified in recent years, the genetic mechanism of schizophrenia is still largely unknown. Recently, pathway-based analysis for GWAS data have constantly emerged and served as a complement for the original analysis of GWAS. Jointly analyzing genes with the same biological pathway simultaneously complements the single-SNP analysis and can reveal new insights to the understanding of complex disorders. Therefore, utilizing the pathway-based analysis may provide us with important information about the pathogenesis and potential treatment of schizophrenia.

In this study, using pathway-based analysis, we identified that the Cell adhesion molecules pathway (hsa04514) might contribute to the schizophrenia susceptibility in Chinese Han population. Moreover, in gene level, 12 genes were identified to be significantly associated with schizophrenia (*P*<0.01). Though the CAMs pathway was associated with schizophrenia in pathway level in both Chinese Han and European population, the significant associated CAMs genes in our dataset were different from those significant CAMs genes (*HLA-DQA1*, *CDH4*, *NRXN1 and CNTNAP2*) reported in European population [17]. There was difference between the gene-based test of these two analyses. We performed the gene-based test using GATES method in our pathway analysis, which could partly alleviate the effect of gene size and LD pattern [34]. However, Hargreaves et al, used the most significant SNP to represent the corresponding gene [17]. In addition, the differences in environmental exposures and genetic background between Chinese and European populations might suggest susceptibility genes of schizophrenia. As shown in S1 Table, the minor allele frequency (MAF) for the allele of associated SNP is much different between European and Chinese populations, according to the HaploReg v3 database (http://www.broadinstitute.org/mammals/haploreg/haploreg_v3.php). Further validation of these CAMs genes in world population needs to be conducted in the future.

Among these 12 significant genes in our dataset, several have suggested to involve in brain function or neurodevelopment psychiatry in previous studies. For example, *MPZ* encodes a major structural protein of peripheral myelin, and an increasing number of evidence supported myelin-related protein and pathways contribute to the risk of schizophrenia and other psychiatry disorders [14, 48]. The dysfunction of *NRCAM* may have an effect on several developmental process including synapse function and the formation of the mature node of Ranvier related to myelin sheath [49, 50]. Furthermore, perturbation of NRCAM function can be associated with psychiatry disorders, containing schizophrenia, autism, Alzheimer's disease, mathematics disability and drug addiction [51]. Genes in MHC region had also been widely reported to be associated with schizophrenia in previous researches [52]. More and more evidence suggested that immune dysfunction might play pivotal roles in schizophrenia. For example, there was evidence for involvement of infection in schizophrenia risk, mostly related to prenatal or early life exposures [53, 54]. Furthermore, increased rates of autoimmune and inflammatory disorders were reported in schizophrenia [55]. Probably, the results presented here provided further evidence that genetic variation involved in immune system influencing schizophrenia susceptibility. Our study might make complement for the association between CAMs pathway and schizophrenia.

Based on the results of single variant analysis and gene-based test from our previous GWAS and PGC, we selected the promising susceptibility gene *NLGN1* to do a fast-track replication analysis. The *NLGN1* gene encodes a member of a family of neuronal cell surface proteins, acting as splice site-specific ligands for beta-neurexins and might be involved in the formation and remodeling of synaptic contacts [56]. NLGN1 protein is special localized on excitatory synapses and showed the coaggregation with PSD-95, which is involved for example in the localization of NMDA2 receptor and K^+^ channels to synapses [57]. *NLGN1* knockout mice exhibited an increase in repetitive, stereotyped grooming behavior, a slight decrease in social interaction and deficits in spatial learning and memory correlated to impaired hippocampal LTP [30]. Finally, several researches have indicated *NLGN1* was significant associated with a variety of psychiatric phenotypes including autism and major recurrent depression [31,32]. Chromosomal partial deletion of *NLGN1* was association with severe intellectual disability, seizures disorder [58]. Lines of genetic evidence and functional studies make *NLGN1* a more promising candidate gene of schizophrenia. Future experiments are required to replicate the association of other significant genes with schizophrenia.

Additionally, in current study, several lines of evidences were also provided to support *NLGN1* as a schizophrenia susceptibility gene. First, several SNPs in *NLGN1* were identified to be associated with schizophrenia in an independent sample. Second, we performed a combined study of this replication sample and the first-stage GWAS sample. The combined study revealed that seven SNPs were still strongly associated with schizophrenia. Third, expression profiling analysis suggested that the *NLGN1* gene preferentially expressed in human brain. Human Brain temporal-spatial expression analysis of *NLGN1* also supported the potential role of *NLGN1* in schizophrenia. Forth, we found a genetic variant (rs1488547) in *NLGN1* that was significantly associated with the mean expression level of *NLGN1* across ten human brain regions and survived after Bonferroni correction. The subjects with GG genotype showed higher expression. In current study, the G allele of rs1488547 was the risk allele. This suggested that higher expression of NLGN1 in brain might be associated with schizophrenia. However, the eQTL database we used was based on European population, and the MAF of rs1488547 is highly different between European and Asian populations (S1 Table). Thus, further studies are warranted to explore the association between rs1488547 and the expression of NLGN1 in Chinese Han population. Taken together, these evidences supported that the gene *NLGN1* has a potential role in pathogenesis of schizophrenia. Thus, further investigation is warranted.

Hence, the pathway-based analysis method could complement standard single marker analysis and contribute to the identification of new genetic factors underlying psychiatric disorders. However, the present study also has several limitations. Based on our previous GWAS and PGC schizophrenia meta-analysis, we selected only one promising gene *NLGN1* to validate in independent samples. The P-values of SNPs in *NLGN1* reported here would not be significant if we corrected for all SNPs across the genome (*P*<5×10^−8^). We did not include enough polymorphic markers to cover *NLGN1*, especially the regulatory elements, for example, the promoter variants that might influence the expression of these genes. Thus, the other associated CAMs genes and more associated polymorphic markers need to be verified in the larger independent samples in the future.

In summary, the association between CAMs pathway and schizophrenia was further validated in Chinese Han population. It was suggested that a crucial role of CAMs pathway in genetic mechanism of schizophrenia. Furthermore, the results indicated that *NLGN1* was associated with schizophrenia in Chinese Han Populations. Further work is warranted to investigate the mechanism underlying the CAMs pathway and *NLGN1* gene.

## Supporting Information

S1 Fig Flow chart of present study. Based on the hypothesis that the CAMs pathway was associated with schizophrenia in Chinese Han population, we combined our previous schizophrenia GWAS and the CAMs pathway from KEGG database with the KGG software tool. Firstly, the SNP was mapped to the corresponding gene in CAMs pathway. Second, KGG combined SNP-based p-values using the Gene-Based Association Test (GATES). Lastly, the pathway-level statistics were calculated by combining all gene-based p-values for association. Then, we focused on the NLGN1 gene as it was identified to be associated with other psychiatric disorders, including autism and bipolar disorder and involved in many important brain functions such as synaptic plasticity and long-term memory. However, to our knowledge, compared with the other associated genes (gene P-value<0.01), the role of NLGN1 for schizophrenia was rarely known. Then, we extracted SNP information from PGC website and found the NLGN1 gene was nominally associated with schizophrenia in both SNP and gene level. To further validate the association of NLGN1 in Chinese Han population, we selected 8 SNPs (*P*<0.01) to validate in a new independent (1,814 schizophrenia cases and 1,457 controls).
(DOCX)

S1 Table The summary results of several top associated SNPs of *NLGN1* in our GWAS data and PGC GWAS data. (DOCX)

S2 Table Haplotype results of the blocks derived from our previous GWAS sample. (DOCX)

S3 Table Haplotype results of the blocks derived from our validated samples (1814 cases and 1487 controls). (DOCX)
