# Supplementary figures and images for: Evidence for Association of Cell Adhesion Molecules Pathway and NLGN1 Polymorphisms with Schizophrenia in Chinese Han Population
Source: PLoS One. 2015 Dec 16;10(12):e0144719. doi: 10.1371/journal.pone.0144719 (PMC4682938; doi:10.1371/journal.pone.0144719)

**S1 Fig. Flow chart of present study.**


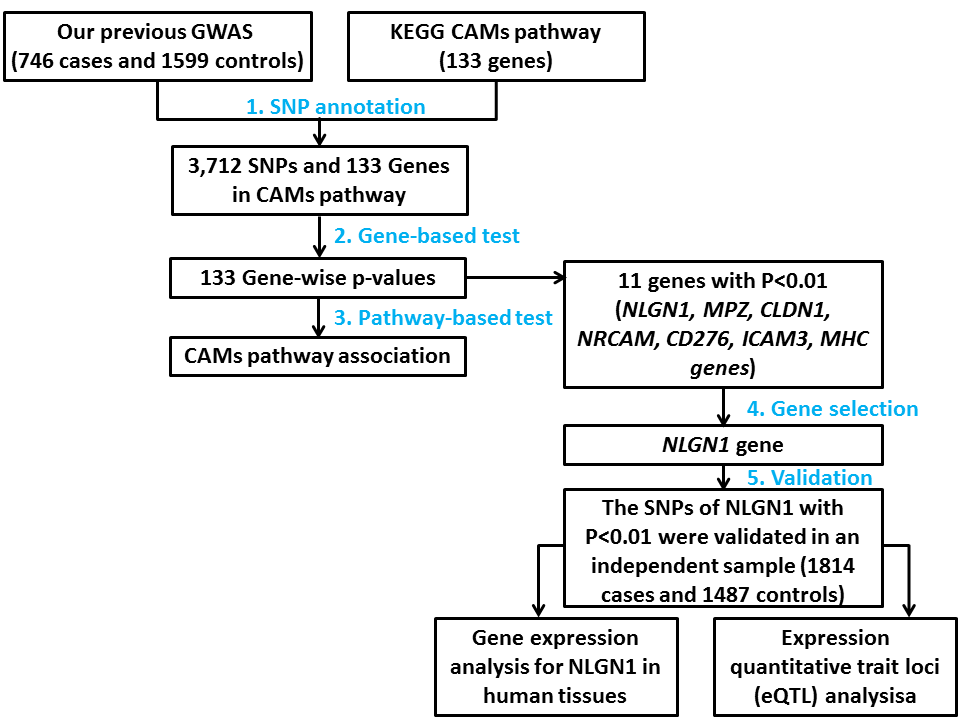

Supplement: S1 Fig — Based on the hypothesis that the CAMs pathway was associated with schizophrenia in Chinese Han population, we combined our previous schizophrenia GWAS and the CAMs pathway from KEGG database with the KGG software tool. Firstly, the SNP was mapped to the corresponding gene in CAMs pathway. Second, KGG combined SNP-based p-values using the Gene-Based Association Test (GATES). Lastly, the pathway-level statistics were calculated by combining all gene-based p-values for association. Then, we focused on the NLGN1 gene as it was identified to be associated with other psychiatric disorders, including autism and bipolar disorder and involved in many important brain functions such as synaptic plasticity and long-term memory. However, to our knowledge, compared with the other associated genes (gene P-value<0.01), the role of NLGN1 for schizophrenia was rarely known. Then, we extracted SNP information from PGC website and found the NLGN1 gene was nominally associated with schizophrenia in both SNP and gene level. To further validate the association of NLGN1 in Chinese Han population, we selected 8 SNPs (P<0.01) to validate in a new independent (1,814 schizophrenia cases and 1,457 controls). (DOCX) [file pone.0144719.s001.docx]
