# Supplementary material for: Evidence for Association of Cell Adhesion Molecules Pathway and NLGN1 Polymorphisms with Schizophrenia in Chinese Han Population
Source: PLoS One. 2015 Dec 16;10(12):e0144719. doi: 10.1371/journal.pone.0144719 (PMC4682938; doi:10.1371/journal.pone.0144719)
Supplement: S1 Table — (DOCX) [file pone.0144719.s002.docx]

**Table S1. The summary results of several top associated SNPs of *NLGN1* in our GWAS data and PGC GWAS data.**

| SNP | Position | A1/A2 | CHN freq | EUR freq | Our GWAS data | | PGC GWAS data | |
| --- | --- | --- | --- | --- | --- | --- | --- | --- |
|  |  |  |  |  | *P* values | OR | *P* values | OR |
| rs13074723 | 173522097 | G/A | 0.23 | 0.71 | 0.0049 | 1.242 | 0.05175 | 0.97677 |
| rs1488547 | 173525768 | A/G | 0.23 | 0.71 | 0.0085 | 1.225 | 0.09398 | 1.02051 |
| rs2861598 | 173544812 | A/G | 0.23 | 0.70 | 0.0035 | 1.253 | 0.165 | 1.01684 |
| rs4280663 | 173623133 | A/G | 0.24 | 0.68 | 0.0011 | 1.289 | 0.3358 | 1.01126 |
| rs4399918 | 173604333 | A/G | 0.42 | 0.12 | 0.0035 | 0.831 | 0.134 | 1.02511 |
| rs4513478 | 173626250 | A/G | 0.43 | 0.72 | 0.0057 | 1.192 | 0.7678 | 1.00351 |
| rs6792822 | 173697708 | C/T | 0.24 | 0.55 | 0.0000718 | 1.32 | 0.4274 | 0.99104 |
| rs34626435 | 173504147 | G/A | 0.03 | 0.12 | N.A. | N.A. | 3.32E-07 | 1.09111 |
| rs71310561 | 173511424 | T/G | 0.03 | 0.13 | N.A. | N.A. | 8.58E-07 | 0.92302 |
| rs2046722 | 173507744 | T/C | 0.03 | 0.13 | N.A. | N.A. | 9.84E-07 | 0.92312 |

A1/A2, minor allele/major allele in Chinese population; CHN freq, the allele frequency of A1 in Chinese population; EUR freq, the allele frequency of A1 in European population; N.A.=Not available; OR, odd ratio.
