# Supplementary material for: Evidence for Association of Cell Adhesion Molecules Pathway and NLGN1 Polymorphisms with Schizophrenia in Chinese Han Population
Source: PLoS One. 2015 Dec 16;10(12):e0144719. doi: 10.1371/journal.pone.0144719 (PMC4682938; doi:10.1371/journal.pone.0144719)
Supplement: S2 Table — (DOCX) [file pone.0144719.s003.docx]

**Table S2. Haplotype results of the blocks derived from our previous GWAS sample.**

| **Haplotype** | **Case ^a^** | **Control ^a^** | ***χ^2^*** | ***P* value ^b^** | **OR (95% CI)** | **Global** | |
| --- | --- | --- | --- | --- | --- | --- | --- |
|  |  |  |  |  |  | ***χ^2^*** | ***P* value ^b^** |
| ***Block1*** | | | | | | | |
| GAA | 0.77 | 0.82 | 14.08 | **0.0002 (0.0137)** | 0.74 (0.63-0.87) | 14.76 | **1.22E-4** |
| AGG | 0.22 | 0.17 | 14.80 | **0.0001 (0.0142)** | 1.36 (1.16-1.59) |  |  |
| ***Block2*** | | | | | | | |
| GGA | 0.42 | 0.46 | 5.25 | 0.022 (0.2381) | 0.862 (0.76-0.98) | 14.41 | **7.43E-4** |
| GAA | 0.36 | 0.37 | 0.22 | 0.6371 (0.9394) | 0.97 (0.85-1.10) |  |  |
| AAG | 0.21 | 0.16 | 13.87 | **0.0002 (0.0024)** | 1.36 (1.16-1.60) |  |  |

^a^ Frequencies of case and controls are shown.

^b^ Permutation P-values are shown in parentheses *P* values, the significance P-values (P<0.05) are bold.

OR, odds ratio; CI, confidence interval.
