# Supplementary material for: Evidence for Association of Cell Adhesion Molecules Pathway and NLGN1 Polymorphisms with Schizophrenia in Chinese Han Population
Source: PLoS One. 2015 Dec 16;10(12):e0144719. doi: 10.1371/journal.pone.0144719 (PMC4682938; doi:10.1371/journal.pone.0144719)
Supplement: S3 Table — (DOCX) [file pone.0144719.s004.docx]

**Table S3. Haplotype results of the blocks derived from our validated samples (1814 cases and 1487 controls).**

| **Haplotype** | **Case ^a^** | **Control ^a^** | ***χ^2^*** | ***P* value ^b^** | **OR (95% CI)** | **Global** | |
| --- | --- | --- | --- | --- | --- | --- | --- |
|  |  |  |  |  |  | ***χ^2^*** | ***P* value ^b^** |
| ***Block1*** | | | | | | | |
| GAA | 0.78 | 0.81 | 9.02 | **0.0027 (0.0122)** | 0.83 (0.73-0.94) | 8.992 | **0.0027** |
| AGG | 0.21 | 0.18 | 8.89 | **0.0029 (0.0127)** | 1.21 (1.07-1.36) |  |  |
| ***Block2*** | | | | | | | |
| GGA | 0.42 | 0.45 | 3.73 | 0.0534 (0.2325) | 0.91 (0.82-1.00) | 12.23 | **0.0022** |
| GAA | 0.35 | 0.36 | 0.56 | 0.4543 (0.9392) | 0.96 (0.87-1.06) |  |  |
| AAG | 0.21 | 0.18 | 12.16 | **0.0005 (0.0029)** | 1.25 (1.10-1.4) |  |  |

^a^ Haplotype frequencies of case and controls.

^b^ Permutation P-values are shown in parentheses, the significance P-values (P<0.05) are bold.

OR, odds ratio; CI, confidence interval.
